# Changes in Nutrient Profile and Antioxidant Activities of Different Fish Soups, Before and After Simulated Gastrointestinal Digestion

**DOI:** 10.3390/molecules23081965

**Published:** 2018-08-06

**Authors:** Gaonan Zhang, Shujian Zheng, Yuqi Feng, Guo Shen, Shanbai Xiong, Hongying Du

**Affiliations:** 1 Key Laboratory of Environment Correlative Dietology, Ministry of Education, College of Food Science and Technology, Huazhong Agricultural University, Wuhan 430070, China; gaonanzhang@outlook.com (G.Z.); 18067911717@163.com (G.S.); xiongsb@hzau.edu.cn (S.X.); 2 Key Laboratory of Analytical Chemistry for Biology and Medicine of the Ministry of Education, Department of Chemistry, Wuhan University, Wuhan 430070, China; 2010301040092@whu.edu.cn (S.Z.); yqfeng@whu.edu.cn (Y.F.); 3 National R & D Branch Center for Conventional Freshwater Fish Processing, Wuhan 430070, China

**Keywords:** fish soup, in vitro simulated gastrointestinal digestion, fatty acid, free amino acids, antioxidant activity

## Abstract

Different kinds of freshwater fish soups show a diverse range of health functions, due to their different nutritional substances and corresponding bioactivities. In the current study, in order to learn the theoretical basis of the potential role fish soup plays in diet therapy functions, the changes of nutrient profiles and antioxidant activities in crucian carp soup and snakehead soup (before and after simulated gastrointestinal digestion) were investigated, such as chemical composition, free amino acids, mineral and fatty acid contents, DPPH radical scavenging activity, ferrous ion chelating activity, hydroxyl radical-scavenging activity and the reducing power effect. Results show that the content of mineral elements in snakehead fish soup was significantly higher than that of crucian carp soup, especially for the contents of Ca, Zn, Fe. The content of total amino acid (TAA) of crucian carp soup (82.51 mg/100 mL) was much higher than that of snakehead fish soup (47.54 mg/100 mL) (*p* < 0.05). Furthermore, the antioxidant capacity of crucian carp soup was stronger than that of snakehead soup. The intensive profiles of nutritional composition and antioxidant activities of these two kinds of fish soups were expected to partly provide the theoretical basis of therapeutic effects.

## 1. Introduction

Soup has different properties and nutrients based on the different materials used during the cooking procedure, including chicken [1], beef [2], pork [3], fish [4], etc. It plays an essential role in physical growth, maintenance of normal bodily functions, and good health. In addition, drinking soup could increase satiety and lower the incidence of obesity by helping people keep fit [5]. Therefore, various soups have become more and more important for people who consume them frequently, especially nutritious and healthy soups. Freshwater fish is rich in nutrition, and it is a source of high-quality proteins, minerals [6] and essential fatty acids, particularly polyunsaturated fatty acids—docosahexaenoic acid (DHA, C22:6n3) and eicosapentaenoic acid (EPA, C20:5n3)—which are good for one’s health [7,8,9] and preventing many coronary artery diseases [7]. Therefore, freshwater fish have the potential to become raw materials for a health preserving soup.

Snakehead (*Channa argus)* and crucian carp (*Carassius auratus*) are two kinds of freshwater fish that are popular in Hubei Province (China) as health-care soup ingredients. Snakehead soup is nutritious, and it is commonly used during adjuvant therapy for people with a weak body and a poor nutritional situation, especially for the healing of wounds and burns [10]. Crucian carp soup has an attractive milky white color and a great taste; it is suitable for the elderly and for lactating women [11]. The nutritional profiles of the fish soups may determine the different dietotherapy functions; therefore, an investigation of nutrient components may provide relevant information.

Furthermore, the biological activity of food is further enhanced by the release of bioactive compounds and peptides during the digestive procedure, thermal pre-treatment, microbial fermentation or other technological processing [12]. Studies of the pharmacological effects of fish peptides or the other metabolites hydrolyzed by digestion have revealed their different functions, such as antihypertensive, immunomodulatory, antioxidant, antitumor, and antimicrobial activities [13]. Antioxidant activities as a representative biological activity were closely to related to the function of the food, it is commonly used to assess or explain the effect of adjuvant therapy of food, even for fish or fish products [14].

In this study, the intensive profiles of nutritional compositions of these two homemade fish soups (crucian carp soup and snakehead soup) were systematically investigated. In addition, the antioxidant activities of the fish soups, before and after simulated gastrointestinal digestion, were also explored. This study could provide the theoretical basis of diet composition and properties for understanding the reason of various medicinal tonic functions of different nourishing soups.

## 2. Results and Discussion

### 2.1. Proximate Chemical Composition

The chemical compositions of the crucian carp soup and snakehead soup are shown in Table 1. The results showed that water was the major component in both freshwater fish soups before and after simulated gastrointestinal digestion due to the soup materials issue. There was almost no difference in the water, ash and fat contents observed in both of the fish soups, except the protein content. In the beginning, the crucian carp soup had twice the soluble protein content compared to the snakehead soup; however, the protein content of the snakehead soup had a large increase after in-vitro gastrointestinal digestion. The protein content increased from 0.82%, 0.41% to 0.91%, 0.70% for the crucian carp soup and the snakehead soup, respectively. The increase in protein content was caused by the addition of enzymes and digestion of insoluble proteins, of which the snakehead soup had higher levels. Furthermore, the fat content of both fish soups underwent a slight decrease after simulated gastrointestinal digestion; the reason was that pancreatin is a digestive enzyme which helped the digestion of fat, ultimately leading to a decrease in the amount of total fat [15].

### 2.2. Mineral Elements Analysis

Minerals usually play a key role in biological processes and metabolism and are considered as nutrient minerals related to specific health benefits [16]. As such, the mineral element contents of the crucian carp soup and snakehead soup before and after in-vitro digestion are collected in Table 2. From the mineral element analysis, the results for major minerals (Na, K, Ca,) and trace minerals (Mg Zn, Fe) indicated that there were high concentrations of K and Mg in both fish soups. Comparing the different fish species, the snakehead soup appears to have a higher mineral content, especially Ca, Zn and Fe. Usually, Zn and Fe are involved in the composition of multiple enzyme active centers and have direct or indirect effects on the synthesis of nucleic acids, proteins and the immune process [17]. Research shows that Fe is involved in hematopoiesis, which is an important component of heme iron in erythrocyte, and easily leads to anemia and other symptoms [18]. Zn deficiency can cause metabolic dysfunction and decreased immune functions, leading to infection by bacteria, viruses and fungi, growth retardation and premature and poor wound healing [19,20]. These reasons may be why snakehead soup is suitable for patients after surgery during the time their wound is healing. After the process of digestion, the levels of Na, Zn and Fe significantly increased (*p* < 0.05). During the pancreatin digestion, NaHCO_3_ and NaOH were used to adjust pH, which perhaps was the major reason leading to the increase of Na. Therefore, it is more reasonable for mineral element analysis that content of Na cannot be taken into account in similar future research works. Protein degradation was lower than that of snakehead soup (6.28 mg/100 mL) promoted by chelating metal groups exposure, thus the levels of Zn and Fe went up.

### 2.3. Free Amino Acids Contents

The mean values (with their standard errors) of the amino acids were set out in Table 3, including amino acid (AA) compositions, the concentrations of total amino acids (TAA), total essential amino acids (TEAA) and flavor amino acids for all kinds of fish soup. 

This was done regardless of the status of the fish soup samples, being before and after digestion processing. From Table 3, it can be seen that the content of flavor amino acids for both fish soups were different, in particular the contents of Asp, Glu, Gly, Ala and Arg in the crucian carp soup were 0.4 mg, 1.88 mg, 35.48 mg, 1.43 mg and 0.1 mg in each 100 mL fish soup, but for the snakehead soup it was 0.33 mg, 0.82 mg, 22.92 mg, 7.04 mg and 0.34 mg, respectively. The total content of flavor amino acids in crucian carp soup (38.93 mg/100 mL) were higher than that of snakehead soup (31.45 mg/100 mL). Flavor amino acids, which belong to the (umami)-taste active amino acids, have been considered as one of the main contributors to the flavor of a soup [21]. That therefore should be the reason that crucian carp soup has a more attractive taste than snakehead soup.

The content of essential amino acids and non-essential amino acids TAA of crucian carp soup (82.51 mg/100 mL) were much higher than that of snakehead soup (47.54 mg/100 mL) (*p* < 0.05), while the TEAA of crucian carp soup (4.44 mg/100 mL) was lower than that of snakehead soup (6.28 mg/100 mL) (*p* < 0.05). After in-vitro gastrointestinal digestion, the variation tendency of most TAA and EAA contained in the two kinds of soups sharply increased (*p* <0.05) except His, Gly, Thr and Glu in the crucian carp soup. In the gastrointestinal tract, the soluble proteins degraded into small peptides and free amino acids under the action of pepsin and pancreatin, which are more beneficial to human absorption. The data obtained for the contents of EAA showed that the amount of Thr in the snakehead soup (5.01 mg/100 mL) was almost five times higher compared to the crucian carp soup (1.05 mg/100 mL) after digestion. Thr is involved in a variety of human metabolism functions known as second or third restricted amino acids [22]. The contents of Val, Ile and Leu in the crucian carp soup were higher than that of snakehead soup whether it was digested or not. The Leu, Ile and Val compose the Branched-Chain Amino Acids (BCAAs) which have unique properties with diverse physiological and metabolic roles and are important in immunological and brain functions [23]. This means crucian carp soup has more potential to enhance immune functions than snakehead soup.

### 2.4. Fatty Acids Profile

Fatty acids profiles (% weight of methyl esters), the sum of total saturated fatty acids (SFA), unsaturated fatty acids (UFA), monounsaturated fatty acids (MUFA) and polyunsaturated fatty acids (PUFA) inherent in the crucian carp soup and the snakehead soup before and after digestion are presented in Table 4. Thirty-two kinds of fatty acids, including twenty SFAs, five MUFAs and seven PUFAs, were characterized in the different kinds of original fish soup samples. Twenty-three of them were detected in the crucian carp soup, the exceptions being caprylic acid, pelargonic acid, 12-methyl tridecanoic acid, heneicosanoic acid, tricosanoic acid, lignoceric acid, nervonic acid, linoleic acid and *cis*-11, 14-eicosadienoic acid. Twenty-six fatty acids were detected in the snakehead soup; the ones not detected were caprylic acid, pelargonic acid, arachidic acid, tricosanoic acid, *cis*-8, 11, 14-eicosatrienoic acid and *cis*-11,14-eicosadienoic acid. After simulated gastrointestinal digestion, six fatty acids, which were not monitored in the original crucian carp soup, appeared. However, two fatty acids, 14-methyl palmitic acid and linolenic acid, disappeared in the snakehead soup. Comparing the fatty acid profiles of fish soup samples before and after simulated gastrointestinal digestion, the sum of SFA and UFA showed almost no change in general, the content of MUFA significantly increased (*p* < 0.05) and the content PUFA significantly went down (*p* < 0.05). The content of UFA for the crucian carp soup and the snakehead soup were 62.26% and 59.35%, respectively. The two major fatty acids were oleic acid and palmitic acid, accounting for 44.08% and 23.56%, 38.42% and 25.31% of the total fatty acids in the crucian carp soup and the snakehead soup, respectively. Usually, oleic acid has the capability of lowering total cholesterol and low-density lipoprotein cholesterol, so it has a reputation of being a ‘safe fatty acid’ [24]. 

Palmitic acid is one of the most important dietary fatty acids and plays a crucial role in the cellular biological functions. It is equipped with functions to provide energy for humans or transmit to other fatty acids through metabolism [25]. 

Snakehead soup contained eicosapentaenoic acid (EPA) 3.02% and docosahexaenoic acid (DHA) 5.80% equal to 8.82% of the total fatty acids, which was higher than that of crucian carp soup (5.34%). EPA is an important and necessary nutritional element which can increase cell viability and neuroprotective effects [26]. 

Commonly known as ‘brain gold′, DHA is crucial to the development of the nervous and visual systems and also for its deficiency during gestation, lactation and early stages of life [27]. In addition, nervonic acid was found in snakehead soup before and after digestion, but it was not detected in crucian carp soup. Generally, nervonic acid is a major long-chain MUFA which was found in the white matter of mammalian brains; it plays an important role in the treatment of psychotic disorders and neurological development [28]. Therefore, snakehead soup may have a better capability in accelerating cell growth and dramatic impacts on brain functions and mental health, compared to crucian carp soup. 

In order to investigate the contribution of fatty acids contained in SFA, MUFA and PUFA in different fish soup samples, the distribution plot of different fatty acid types was collected. On the whole, fatty acids existed in both of the freshwater fish soups and rose after simulated gastrointestinal digestion. More specifically, there were 14 SFA (37.6%), four MUFA (54.3%) and five PUFA (7.9%) detected in crucian carp soup. However, after digestion, 20 SFA (37.2%), five MUFA (57.1%) and six PUFA (5.6%) were found in crucian carp soup. For snakehead soup, there were 16 SFA (40.7%), five MUFA (48.3%) and five PUFA (11.0%) identified, after pepsin-pancreatin digestion, there were 17 SFA (40.2%), five MUFA (53.3%) and five PUFA (6.2%). Based on Table 4, the highest content of saturated fatty acids was palmitic acid, which reached 25.1% and 23.6% for crucian carp soup and snakehead soup, respectively.

### 2.5. Antioxidant Activities

#### 2.5.1. DPPH Radical-Scavenging Activity

**Figure 1:**
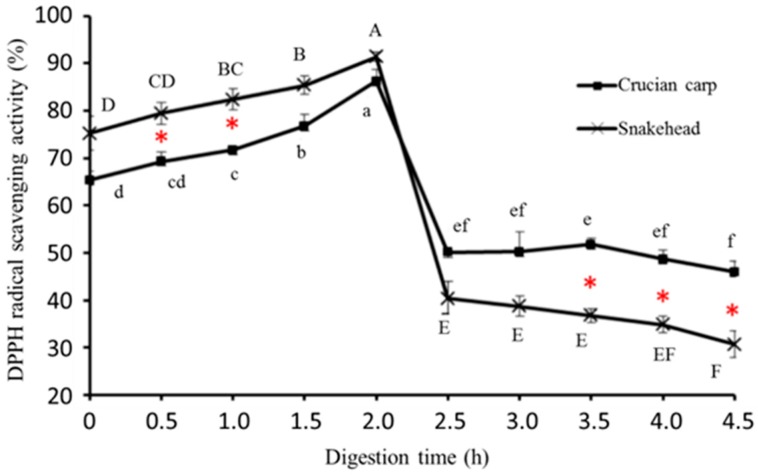
DPPH·radical scavenging activity of crucian carp soup and snakehead soup during simulated gastrointestinal digestion. Note: Different letters represented significant difference at *p* < 0.05; Capital: Comparison in the snakehead group; lowercase: Comparison in the crucian carp; red star: Significant changes between crucian carp soup and snakehead soup under the same digestion time (*p* < 0.05).

DPPH is a widely used stable free radical scavenger or hydrogen donor for assessing antioxidant activities of bioactive compounds and food itself [29]. In this study, The DPPH free radical scavenging activities of two kinds of freshwater fish soups with time during in vitro digestion are shown in Figure 1. 

Before in-vitro digestion, the DPPH radical scavenging activity of snakehead soup was about 75.24%, which was higher than that of crucian carp soup (65.31%); that means the fish soup had DPPH radical-scavenging activity to some extent without any digestion processing. The reason may be that some bioactive compounds, which contained hydrophobic groups and easily capture the DPPH radical, existed in the fish soup. During pepsin digestion, the DPPH radical scavenging activity showed a significant increasing trend in both crucian carp soup and snakehead soup; they separately rose by 32% and 21%. Such results may be attributed to higher exposure to hydrophobic amino acid residues in the peptide chains, which can increase antioxidative properties when some soluble proteins existing in the fish soup were further hydrolyzed by the pepsin. However, from Figure 1 it is clearly seen that both fish soup samples exhibited dramatic decreases in DPPH radical scavenging activities during pancreatin digestion (*p* < 0.05). During the pancreatin digestion stage, the original soluble peptides contained in fish soup were thoroughly hydrolyzed into shorter peptides (three peptides or four peptides) and even amino acids, which have strong hydrophilicity to decrease the DPPH free radical trapping capability; this phenomenon is agreed on in former research [30].

#### 2.5.2. Hydroxyl Radical-Scavenging Activity

**Figure 2:**
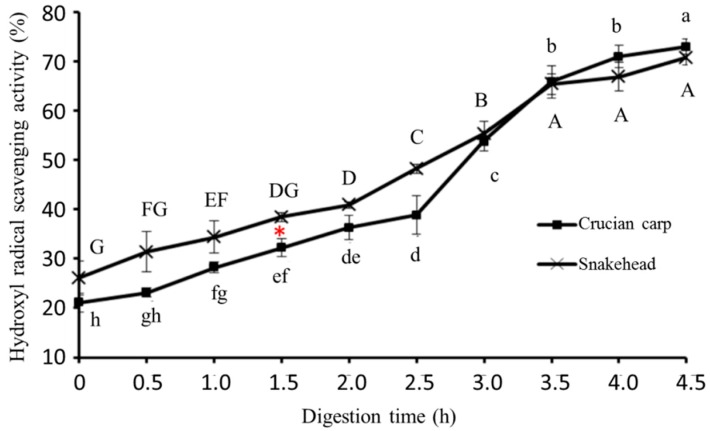
Hydroxyl radical-scavenging activity of crucian carp soup and snakehead soup during simulated gastrointestinal digestion. Note: Different letters represented significant difference at *p* < 0.05; Capital: Comparison in the snakehead group; lowercase: Comparison in the crucian carp; red star: Significant changes between crucian carp soup and snakehead soup under the same digestion time (*p* < 0.05).

Oxidation and metabolism continuously produce various reactive oxygen radicals in the process of an organism’s life activities. However, the hydroxyl radical has a high toxicity to the organism [30]. Reactive oxygen species inducing DNA damage are considered to be one of the major reasons for aging [31]. Therefore, the hydroxyl radical scavenging capacity is usually used to measure the antioxidant activity of the product. As illustrated in Figure 2, there was no significant difference in the hydroxyl radical scavenging activity between crucian carp soup and snakehead soup during in-vitro simulating gastrointestinal digestion. Generally, the ability of the hydroxyl radical scavenging activity shown in both fish soups kept rising during digestion, although the hydroxyl radical scavenging activity of the snakehead soup was higher than that of the crucian carp soup. After finishing the simulated Gastrointestinal digestion, the value of the hydroxyl radical scavenging activity reached 3.6 and 2.7 times the original values for crucian carp and snakehead soup, respectively. The reason may be that more peptides were generated in fish soups during the simulated gastrointestinal digestion process, this led to more effective hydrogen or electron donors to capture hydroxyl radical, which supports previously published works [32].

#### 2.5.3. Ferrous Ion Chelating Activity

**Figure 3:**
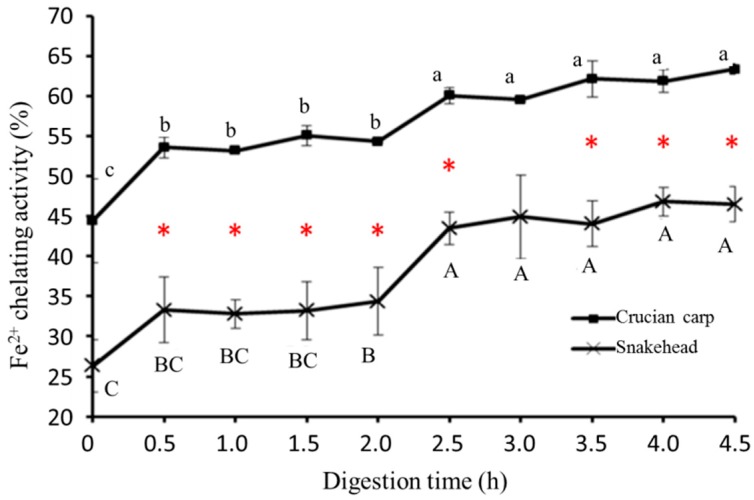
Ferrous ion chelating activity of crucian carp soup and snakehead soup during simulated gastrointestinal digestion process. Note: Different letters represented significant difference at *p* < 0.05; Capital: Comparison in the snakehead group; lowercase: Comparison in the crucian carp; red star: Significant changes between crucian carp soup and snakehead soup under the same digestion time (*p* < 0.05).

Fe^2+^ chelation might render important antioxidative effects by impeding metal-catalyzed oxidation [33]. Therefore, Fe^2+^ chelating activity was also measured in this study to assess the free radical scavenging activity of fish soup samples comprehensively. The Fe^2+^ chelating activity of the two kinds of freshwater fish soups during in vitro digestion was shown in Figure 3. It can be seen that there was a significant change to the metal chelating activity of the two kinds of fish soups (*p* < 0.05) within the first half hour of the digestion procedure. For crucian carp soup, the Fe^2+^ chelating activity rose to 53.58 ± 1.33% from 44.43 ± 5.2% in the pepsin stage and went up to 60.05 ± 1.04% from 54.3 ± 0.11% in the pancreatin stage. For snakehead soup, the value of metal chelating activity increased from 26.32 ± 3.3% to 33.29 ± 4.07% in the pepsin stage and from 34.37 ± 4.2% to 43.48 ± 1.98% in the pancreatin stage. It could be inferred that, during the whole digestion, more free amino acids were released, so more metal chelating groups were exposed or generated to bind Fe^2+,^ which led to the increase in the antioxidant activity of fish soup.

#### 2.5.4. Reducing Power

**Figure 4:**
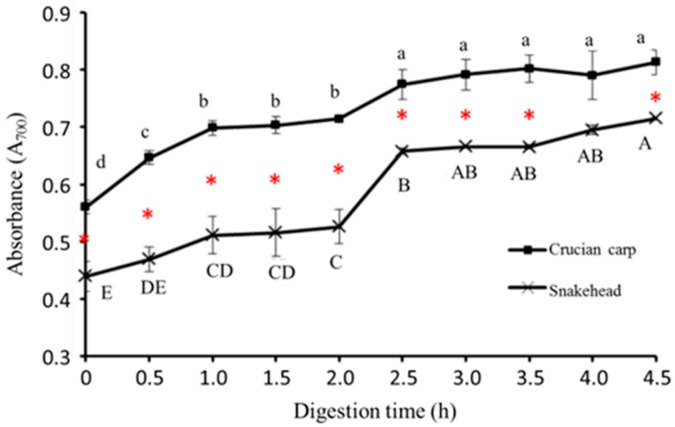
Reducing power of crucian carp soup and snakehead soup during simulated gastrointestinal digestion process. Note: Different letters represented significant difference at *p* < 0.05; Capital: Comparison in the snakehead group; lowercase: Comparison in the crucian carp; red star: Significant changes between crucian carp soup and snakehead soup under the same digestion time (*p* < 0.05).

The reducing power activity, which may serve as a significant reflection of antioxidant activity, was determined using a modified Fe(III) to Fe(II) reduction assay; the yellow color of the test solution changes to various shades of green and blue depending on the reducing power of the samples. The presence of antioxidants in the samples causes the reduction of the Fe^3+^/Ferricyanide complex to the ferrous form. Therefore, Fe^2+^ can be monitored by measuring of the formation of Perls Prussian blue at 700 nm [34]. The reducing power of the two kinds of fish soups is shown in Figure 4; it is very clear that the reducing power of crucian carp soup is much stronger than that of snakehead soup, whether it was processed by simulated gastrointestinal digestion or not. Particularly, reducing power of both fish soups increased within the first hour (*p* < 0.05), then it kept the invariant mode from one hour to two hours during the pepsin digestion step. In the simulated intestinal digestion step, there appeared to be a dramatic increase in reducing power within half an hour after 2 h of the whole digestion time. After that, the reducing power of fish soups seemed to stay stable for the remaining time. Generally, the tendency of reducing power in these two kinds of fish soups was consistent with the result of the Fe^2+^ chelating activity.

## 3. Statistical Analysis

All experiments were taken in triplicate, and all analyses were also conducted in triplicate. Statistical analysis was performed with MS Excel (Microsoft Windows 2010, Redmond, WA, USA) and SAS version 9.2 (SAS Institute Inc., Cary, NC, USA). For statistical analysis, one-way analyses of variance (ANOVA) were employed to determine the significance of differences and followed by least significant difference (LSD) post hoc tests to examine the differences among groups. The criterion for statistical significance was a probability value of 0.05 (*p* < 0.05). Data was expressed as the mean ± standard deviation (SD).

## 4. Materials and Methods

### 4.1. Materials and Reagents

Fresh snakehead (*Channa argus)* (~750 g) and crucian carp (*Carassius auratus*) (~250 g) were purchased from a local market in Huazhong Agricultural University, Wuhan, Hubei, China. Each specimen was gutted and cleaned. All animal (fish) procedures were performed in accordance with the Guidelines for Care and Use of Laboratory Animals of Huazhong Agricultural University and Experiment were approved by the Animal Care and Use Committee of Huazhong Agricultural University (Approval number: 201603060088). All chemicals used in this work were analytical grade.

### 4.2. Preparation of Fish Soup Samples

According to the method of Tang [35], the handled fish was cooked at a suitable mince/solution ratio of 1:4 (*w/v*) adopting a stew soup recipe with an induction cooker (RT2134, Midea, Foshan, Guangdong, China) for 1.5 h. At the beginning the power was set to 500 W to simmer the soup for 20 min, then the power was kept at 300 W and the soup maintained at boiling. Then the soup was filtered with six layers normal gauze to wipe out the solid-state impurities. Then the filtered fish soup samples were used for further analysis.

### 4.3. Simulated Gastrointestinal Digestion

A two-step process was used to simulate the gastric and intestinal digestion of fish soup using the in-vitro enzymatic digestion protocol described in Zheng et al. [36] with slight modification. The pH of the samples was adjusted to 2.0 with 1 M HCl, then pepsin was added (4%, *w/w*, protein basis). The mixture was incubated at 37 °C for 2 h in a shaking water bath. Subsequently, the pH value was adjusted back to 5.3 with 0.9 M NaHCO_3_ and further to 7.5 with 1 M NaOH. Then pancreatin was added (5%, *w/w*, protein basis) and the mixture was further incubated at 37 °C for 2.5 h. After incubation, the test tubes were kept in a boiling water bath for 10 min to inactivate the enzyme.

To analyze the antioxidant activities during the process of digestion, the samples were collected at different time intervals (0 h, 0.5 h, 1.0 h, 1.5 h, 2.0 h, 2.5 h, 3.0 h, 3.5 h, 4.0 h, 4.5 h) during in-vitro gastrointestinal digestion. All samples were adjusted to pH 7.0 and treated with 10% trichloroacetic acid (TCA) and centrifuged at 12,000 rpm for 20 min. After centrifugation, the supernatant (protein hydrolysate) was subpackaged into 50 mL centrifuge tubes and stored at −20 °C for further usage. Both kinds of fish soups (raw fish soup and in vitro simulated gastrointestinal digestion) were analyzed in the following week. 

### 4.4. Proximate Chemical Composition

Before the chemical composition analysis, the filtered fish soups before or after in vitro simulated gastrointestinal digestion were centrifuged at 5,000 rpm (Avanti J-26 XP, Beckman Coulter, Atlanta, GA, USA). The proximate composition was performed on samples using the standard methods (AOAC 1996) [37]. The moisture content of the samples was determined by drying samples in an oven at 105 °C for 16–18 h (AOAC Method 950.46) [37]. The total protein was determined using the Kjeldahl method (AOAC Method 940.25) [37]. During the analysis, an automated distillation unit (Bűchi 339, Postfach, Switzerland) was utilized, and a factor of 6.25 was used to convert the nitrogen content into the protein content. Ash was determined by the incineration of samples in a muffle furnace at 550 °C for 18 h (AOAC Method 938.08) [37]. The extraction of total fat was performed according to the method of Folch et al [38].

### 4.5. Mineral Element Analysis

Sample preparation and determination of the mineral contents were according to Jiang et al. [39]. The soup sample (20 g) was weighed and placed into a crucible, and then carbonized at 250 °C on an electrothermal plate until the sample was fully black. The crucibles with samples were dry-ashed by heating them in a muffle furnace at 550 °C (about 10−12 h). Then the sample was incinerated, and a white residue was obtained, which was carefully transferred into a 50 mL volumetric flask, dissolved with 5 mL of 6 M HCl and then diluted to 50 mL with water. The mineral contents (K, Ca, Na, Mg, Fe, Zn) of the fish soups were detected using an Atomic Absorption Spectrophotometer (TAS-990F, Fairburn, Shanghai, China).

### 4.6. Free Amino Acid Analysis

Free amino acids (FAA) were extracted from the fish soups according to Tanimoto′s method [40] with minor modifications. The fish samples were mixed in the same volume of sulfosalicylic acid and the mixture was centrifuged at 3000 r/min for 15 min, then the supernatant was collected. 1 mL supernatant solution was mixed with 6 mL TCA and centrifuged for 15 min, and the supernatant was transferred to the round bottom flask. The rotary evaporator was used, 10 mL distilled water was added, and this was repeated several times to completely remove TCA. In the end, the extracted solution was mixed with 0.02 M HCl and the pH value was adjusted to 2.0. The prepared samples were analyzed using an L-8900 Amino Acid Analyzer (Hitachi, Tokyo, Japan).

### 4.7. Fatty Acid Analysis

The lipid extraction and the fatty acid methyl esters (FAMEs) were gradually prepared with the approaches of Floch et al [38] and Hartman and Lago [41] with slight modifications. The detailed steps were as follows: the fish soup sample (~5 mL) was mixed with 30 mL chloroform-methanol (2:1, *v/v*) and was homogenized at 8000 rpm for 20 s twice using T18 digital Ultra-turrax (IKA, Germany). Then the mixture was put under stationary conditions for 1 h, and then filtered after adding 0.2 times the volume of physiological saline. The suspension was centrifuged (3000 rpm, 15 min), the upper liquid was removed and the remaining liquid concentrated using nitrogen purging. Afterwards, 14% BF_3_-methanol reagent (2 mL) was added, and the mixture was kept at 60 °C for 60 min. After cooling, 1 mL of water and 2 mL of hexane were added and the mixture was shaken for about 15 s, and FAMEs were extracted into hexane. Then the top concentrated FAMEs hexane solution was transferred into a tube to be preserved at −20 °C for further analysis. 

A gas chromatography-mass spectrometer (QP2010, Shimadzu, Kyoto, Japan) equipped with a 30 m column (Rtx-5MS column 30 m × 0.25 mm × 0.25 μm column) was used. The GC conditions were set as follows: the oven temperature was initially set at 40 °C for 2 min and then increased to 100 °C at 10 °C/min. The temperature was then increased to 290 °C at 5 °C/min and held for 10 min. Split injection was conducted with a split ratio of 100:1, the flow-rate 1 mL/min; Helium was used as a carrier gas; injector temperature was 250 °C. The MS detection conditions were as follows: Ionization mode, EI^+^; electron energy, 70 eV; full scan acquisition mode; mass range, 45–450 amu.

### 4.8. Measurement of Antioxidant Activity

#### 4.8.1. 1,1-Diphenyl-2-picrylhydrazyl (DPPH) Radical-Scavenging Activity

The DPPH scavenging activities of samples were measured as previously described with slight modifications [42]. In the current study, a 4.0 mL sample was mixed with 1.0 mL DPPH solution (0.1 mM in 99.7% methanol). The mixture was shaken and left for 30 min at room temperature, and the absorbance of the resulting solution was measured at 517 nm using a UV-1750 spectrophotometer (Shimadzu, Kyoto, Japan). Ethanol instead of DPPH was used as the blank, while distilled water was used for the control. The DPPH radical scavenging activity was calculated with the following equation:

DPPH scavenging activity (%) = [1 − (A_sample_ − A_blank_)/A_control_] × 100 (1)

where A_sample_, A_blank_ and A_control_ are the absorbance of sample, blank and control, respectively.

#### 4.8.2. Hydroxyl Radical-Scavenging Activity

The hydroxyl radical scavenging assay was carried out according to the method described previously [43] with some modifications. A reaction mixture solution containing 1,10-phenanthroline (0.75 mM, 1 mL), FeSO_4_ (0.75 mM, 1 mL) and phosphate buffer (pH 7.4, 2 mL) was mixed with a 2 mL sample (2 mL distilled water was used as the control). H_2_O_2_ (1 mL, 0.12%) was added to the mixture and incubated at 37 °C for 60 min, and the absorbance was measured at 536 nm. The results were determined using the following equation:

Hydroxyl radical scavenging activity (%) = [(As − A_1_)/(A_0_ − A_1_)] × 100 (2)

where As is the absorbance of the sample mixture; A_1_ is the absorbance of the control (distilled water instead of sample); and A_0_ is the absorbance of the blank solution containing 1,10-phenanthroline and FeSO_4_.

#### 4.8.3. Fe^2+^ Chelating Activity

The chelation activity on Fe^2+^ was measured based on the following method. An aliquot of 1.0 mL of sample was mixed with 3.7 mL of distilled water, 0.1 mL of 2 mM FeCl_2_ and 0.2 mL of 5 mM ferrozine. The mixture reacted for 20 min at room temperature. Then the absorbance was measured at 562 nm. The distilled water was used as the control, while the distilled water instead of FeCl_2_ and ferrozine was used for the blank. The chelating activity was calculated as follows:

Metal ion chelating activity (%) = (A_control_ − (A_sample_ − A_blank_))/A_control_ × 100 (3)

where A_sample_, A_blank_ and A_control_ are the absorbance of sample, blank and control, respectively.

#### 4.8.4. Reducing Power

The reducing power was measured by using a modified version of Oyaizu’s method [44]. 2 mL sample and 2 mL phosphate buffer (0.2 M, pH 6.6) were mixed with 2 mL 1% K_3_Fe(CN)_6_. The mixture was incubated at 50 °C for 20 min and mixed with 2 mL 10% TCA, followed by centrifugation at 3000 rpm for 10 min. Then, 2 mL of the supernatant were drawn and mixed with 2 mL distilled water, followed by the addition of 0.4 mL of 0.1% FeCl_3_. After 10 min of incubation at room temperature, the absorbance of the resulting Prussian blue solution was read at 700 nm.

## 5. Conclusions

Various foods usually show dietary therapy functions closely related to their individual unique profiles of chemical components, nutrient compositions and bioactivities. In this study, the nutritional compositions and antioxidant activities of two kinds of freshwater fish soup (crucian carp soup and snakehead soup) with different dietotherapy functions were investigated. Changes of the nutritional composition in the fish soup samples before and after in vitro simulated gastrointestinal digestion (pepsin and pancreatin hydrolysis) were also considered. Results showed that the content of the total free amino acids of crucian carp soup were higher than that of snakehead soup, especially for the flavor or umami amino acids content; the dominant amino acids were glutamine and glycine. After simulated gastrointestinal digestion, the number of fatty acids present in both fish soups increased, and snakehead soup had more mineral contents than that of the crucian carp soup. Furthermore, crucian carp soup showed a better antioxidant capacity compared to snakehead soup in the hydroxyl radical-scavenging activity, ferrous ion chelating activity and reducing power. The intensive profiles of nutritional compositions and antioxidant activities of these two kinds of fish soups were expected to partly provide the theoretical basis of therapeutic effects.

## Figures and Tables

**Table 1:** Proximate chemical compositions of different fish soup samples before and after simulated gastrointestinal digestion (g/100 g) (mean ± SD).

Component	Crucian Carp Soup		Snakehead Soup	
	Before	After	Before	After
Moisture	98.82 ± 0.10 ^b^	98.2 ± 0.25 ^c^	99.16 ± 0.12 ^a^	98.64 ± 0.28 ^b^
Ash	0.14 ± 0.01 ^c^	0.095 ± 0.00 ^d^	0.34 ± 0.01 ^b^	0.52 ± 0.02 ^a^
Protein	0.82 ± 0.11 ^b^	0.91 ± 0.05 ^a^	0.41 ± 0.02 ^d^	0.70 ± 0.01 ^c^
Fat	0.18 ± 0.06 ^a^	0.17 ± 0.01 ^b^	0.15 ± 0.09 ^c^	0.13 ± 0.01 ^d^

Note: Different lowercase letters in the same line indicate significant difference (*p* < 0.05).

**Table 2:** Mineral contents of different fish soup samples before and after simulated gastrointestinal digestion (mean ± SD).

Mineral Element	Crucian Carp Soup		Snakehead Soup	
	Before	After	Before	After
Na (mg/g)	0.13 ± 0.03 ^c^	0.69 ± 0.01 ^a^	0.21 ± 0.09 ^b^	0.69 ± 0.00 ^a^
K (mg/g)	0.79 ± 0.05 ^a^	0.79 ± 0.04 ^a^	0.75 ± 0.01 ^a^	0.87 ± 0.04 ^a^
Ca (μg/g)	0.13 ± 0.01 ^b^	0.14 ± 0.01 ^b^	0.81 ± 0.01 ^a^	0.82 ± 0.00 ^a^
Mg (μg/g)	6.12 ± 0.497 ^b^	6.12 ± 0.21 ^b^	6.45 ± 0.165 ^a^	6.01 ± 0.11 ^c^
Zn (μg/g)	0.07 ± 0.01 ^d^	0.16 ± 0.00 ^c^	0.25 ± 0.12 ^b^	0.37 ± 0.01 ^a^
Fe (μg/g)	0.12 ± 0.01 ^d^	0.17 ± 0.00 ^c^	0.35 ± 0.02 ^b^	0.39 ± 0.01 ^a^

Note: Different lowercase letters in the same line indicate significant difference (*p* < 0.05).

**Table 3:** Free amino acid compositions of different fish soup samples before and after simulated gastrointestinal digestion (mg/100mL) (mean ± SD).

Amino Acid	Crucian Carp Soup		Snakehead Soup	
	Before	After	Before	After
Asp ^#^	0.04 ± 0.01 ^d^	1.13 ± 0.01 ^a^	0.33 ± 0.01 ^c^	0.81 ± 0.01 ^b^
Thr *	1.10 ± 0.02 ^c^	1.05 ± 0.05 ^c^	2.04 ± 0.03 ^b^	5.01 ± 0.04 ^a^
Ser	0.66 ± 0.02 ^c^	1.43 ± 0.06 ^b^	0.54 ± 0.01 ^c^	1.87 ± 0.01 ^a^
Glu ^#^	1.88 ± 0.01 ^a^	2.00 ± 0.08 ^a^	0.82 ± 0.06 ^b^	1.99 ± 0.01 ^a^
Gly ^#^	35.48 ± 0.40 ^a^	28.75 ± 1.18 ^b^	22.92 ± 0.44 ^c^	21.44 ± 0.15 ^c^
Ala ^#^	1.43 ± 0.01 ^d^	4 ± 0.18 ^c^	7.04 ± 0.18 ^b^	9.58 ± 0.22 ^a^
Cys	0.66 ± 0.06 ^c^	1.3 ± 0.07 ^b^	0.3 ± 0.22 ^d^	1.63 ± 0.10 ^a^
Val *	0.62 ± 0.01 ^b^	2.7 ± 0.13 ^a^	0.45 ± 0.01 ^b^	3.07 ± 0.01 ^a^
Met	0.52 ± 0.01 ^c^	1.16 ± 0.07 ^b^	0.27 ± 0.01 ^d^	1.57 ± 0.02 ^a^
Ile *	0.27 ± 0.01 ^c^	1.45 ± 0.07 ^a^	0.16 ± 0.02 ^c^	1.27 ± 0.01 ^a^
Leu *	0.96 ± 0.05 ^c^	14.42 ± 0.60 ^a^	0.58 ± 0.01 ^d^	10.69 ± 0.10 ^b^
Tyr	1.88 ± 0.04 ^b^	5.33 ± 0.23 ^a^	0.64 ± 0.21 ^c^	5.83 ± 0.23 ^a^
Phe *	0.68 ± 0.09 ^b^	15.69 ±0.67 ^a^	0.82 ± 0.12 ^b^	15.53 ± 0.08 ^a^
Lys *	0.81 ± 0.01 ^c^	22.07 ± 0.88 ^a^	2.23 ± 0.06 ^b^	20.31 ± 0.12 ^a^
Pro	0.84 ± 0.03 ^b^	0.83 ± 0.21 ^b^	1.16 ± 0.01 ^a^	1.07 ± 0.13 ^a^
His	34.58 ± 0.45 ^a^	29.09 ± 1.22 ^b^	6.9 ± 0.09 ^c^	6.58 ± 0.04 ^c^
Arg ^#^	0.10 ± 0.02 ^d^	18.68 ± 0.72 ^b^	0.34 ± 0.01 ^c^	25.45 ± 0.21 ^a^
Total Amino acid (TAA)	82.51 ± 1.12 ^c^	146.87 ± 0.00 ^a^	47.54 ± 1.63 ^d^	133.13 ± 0.00 ^b^
Total Essential Amino acid (TEAA)	4.44 ± 0.03 ^c^	58.52 ± 2.49 ^a^	6.28 ± 0.02 ^b^	57.42 ± 0.37 ^a^
EAA/TAA	5.38%	39.84%	13.21%	41.96%

Note: Different lowercase letters in the same line indicate significant difference (*p* < 0.05). *: Essential amino acid. ^#^: Flavor amino acid.

**Table 4:** Fatty acid compositions of different fish soup samples before and after simulated gastrointestinal digestion (relation percentage/%) (mean ± SD).

Fatty Acid	Molecular Formula	Crucian Carp Soup		Snakehead Fish Soup	
		Before	After	Before	After
2-Methylbutyric acid	C4:0	0.02 ± 0.00 ^b^	0.03 ± 0.00 ^b^	0.03 ± 0.00 ^b^	0.05 ± 0.01 ^a^
Caproic acid	C6:0	0.01 ± 0.00 ^a^	0.05 ± 0.01 ^b^	0.01 ± 0.00 ^b^	0.01 ± 0.00 ^b^
Caprylic acid	C8:0	/	0.03±0.01	/	/
Pelargonic acid	C9:0	/	0.06±0.01	/	/
Anchoic acid	C9:0	0.10 ± 0.01 ^b^	0.37 ± 0.02 ^a^	0.12 ± 0.02 ^b^	0.04 ± 0.00 ^b^
Lauric acid	C12:0	0.15 ± 0.02 ^b^	0.4 ± 0.02 ^a^	0.16 ± 0.04 ^b^	0.14 ± 0.01 ^b^
Tridecanoic acid	C13:0	0.05 ± 0.01 ^b^	0.08 ± 0.01 ^a^	0.10 ± 0.03 ^a^	0.09 ± 0.01 ^a^
12-Methyl-tridecanoic acid	C13:0	/	0.06 ± 0.00 ^a^	0.10 ± 0.02 ^a^	0.07 ± 0.01 ^a^
Myristic acid	C14:0	0.13 ± 0.01 ^c^	2.57 ± 0.21 ^b^	0.15 ± 0.04 ^c^	4.19 ± 0.29 ^a^
Pentadecanoic acid	C15:0	0.61 ± 0.03 ^b^	0.85 ± 0.03 ^a^	0.62 ± 0.05 ^b^	0.92 ± 0.05 ^a^
Palmitic acid	C16:0	23.56 ± 0.43 ^b^	21.47 ± 0.54 ^c^	25.31 ± 0.64 ^a^	23.42 ± 1.48 ^b^
14-Methylpalmitic acid	C16:0	0.13 ± 0.02 ^b^	1.3 ± 0.1 ^a^	0.20 ± 0.02 ^b^	/
Heptadecanoic acid	C17:0	0.46 ± 0.05 ^c^	0.69 ± 0.01 ^b^	0.73 ± 0.07 ^b^	0.8 ± 0.02 ^a^
Stearic acid	C18:0	7.22 ± 0.05 ^c^	8.15 ± 0.15 ^b^	8.61 ± 0.36 ^b^	9.48 ± 0.72 ^a^
Nonadecanoic acid	C19:0	0.15 ± 0.01 ^b^	0.14 ± 0.01 ^b^	0.12 ± 0.03 ^b^	0.22 ± 0.01 ^a^
Arachidic acid	C20:0	0.36 ± 0.04 ^b^	0.35 ± 0.01^b^	/	0.46 ± 0.01 ^a^
Heneicosanoic acid	C21:0	/	0.4 ± 0.02 ^a^	0.18 ± 0.04 ^b^	0.04 ± 0.01 ^c^
Behenic acid	C22:0	0.10 ± 0.01 ^b^	0.13 ± 0.02 ^b^	0.06 ± 0.01 ^c^	0.18 ± 0.01 ^a^
Tricosanoic acid	C23:0	/	0.02 ± 0.01 ^a^	/	0.03 ± 0.00 ^a^
Lignoceric acid	C24:0	/	0.03 ± 0.01 ^a^	0.06 ± 0.01 ^a^	0.07 ± 0.01 ^a^
Saturated fatty acid (SFA)		37.60 ± 0.77 ^b^	37.17 ± 0.79 ^b^	40.73 ± 0.43 ^a^	40.21 ± 0.5 ^a^
Myristoleic acid	C14:1	0.13 ± 0.01 ^b^	0.11 ± 0.02 ^c^	0.15 ± 0.04 ^b^	0.18 ± 0.02 ^a^
Palmitoleic acid	C16:1	9.39 ± 0.15 ^a^	7.59 ± 0.24 ^b^	9.57 ± 0.44 ^a^	9.07 ± 0.24 ^a^
Oleic acid	C18:1n9	44.08 ± 0.08 ^b^	48.42 ± 1.22 ^a^	38.42 ± 0.33 ^c^	42.81 ± 0.57 ^b^
Erucic acid	C22:1	0.62 ± 0.03 ^b^	0.98 ± 0.06 ^a^	0.14 ± 0.02 ^c^	1.15 ± 0.07 ^a^
Nervonic acid	C24:1	/	0.01 ± 0.00 ^b^	0.07 ± 0.02 ^a^	0.08 ± 0.02 ^a^
Monounsaturated fatty acid (MUFA)		54.34 ± 0.22 ^b^	57.12 ± 1.07 ^a^	48.34 ± 0.83 ^c^	53.29 ± 0.31 ^b^
Linoleic acid	C18:2n6	/	0.42±0.03 ^a^	0.14 ± 0.03 ^b^	0.06 ± 0.01 ^c^
Arachidonic acid	C20:4n6	1.52 ± 0.03 ^a^	1.25 ± 0.08 ^b^	1.83 ± 0.10 ^a^	1.11 ± 0.14 ^b^
*Cis*-5,8,11,14,17-Eicosapentaenoic acid	C20:5n3	2.04 ± 0.06 ^b^	1.26 ± 0.06 ^c^	3.02 ± 0.09 ^a^	1.61 ± 0.19 ^c^
*Cis*-8,11,14-Eicosatrienoic acid	C20:3	0.45 ± 0.03 ^a^	0.66 ± 0.09 ^a^	/	/
Linolenic acid	C18:3	0.61 ± 0.01 ^a^	0.73 ± 0.0^1 a^	0.21±0.03 ^b^	/
*Cis*-11,14-Eicosadienoic acid	C20:2	/	/	/	0.03 ± 0.00
Docosahexenoic acid	C22:6n3	3.30 ± 0.03 ^b^	1.33 ± 0.12 ^c^	5.80 ± 0.27 ^a^	3.41 ± 0.22 ^b^
Polyunsaturated fatty acids (PUFA)		7.92 ± 0.12 ^b^	5.64 ± 0.08 ^d^	11.01 ± 0.12 ^a^	6.23 ± 0.23 ^c^
Unsaturated fatty acid (UFA)		62.26 ± 0.34 ^a^	62.76 ± 1.01 ^a^	59.35 ± 0.71 ^b^	59.52 ± 0.30 ^b^

Note: Different letters represented significant difference at *p* < 0.05.
